# The Role of Complement Inhibition in Thrombotic Angiopathies and Antiphospholipid Syndrome

**DOI:** 10.4274/tjh.2015.0197

**Published:** 2016-02-17

**Authors:** Doruk Erkan, Jane E. Salmon

**Affiliations:** 1 Hospital for Special Surgery, Weill Cornell Medicine, New York, United States

**Keywords:** Antiphospholipid syndrome, Complement inhibition, eculizumab, Thrombotic angiopathy

## Abstract

Antiphospholipid syndrome (APS) is characterized by thrombosis (arterial, venous, small vessel) and/or pregnancy morbidity occurring in patients with persistently positive antiphospholipid antibodies (aPL). Catastrophic APS is the most severe form of the disease, characterized by multiple organ thromboses occurring in a short period and commonly associated with thrombotic microangiopathy (TMA). Similar to patients with complement regulatory gene mutations developing TMA, increased complement activation on endothelial cells plays a role in hypercoagulability in aPL-positive patients. In mouse models of APS, activation of the complement is required and interaction of complement (C) 5a with its receptor C5aR leads to aPL-induced inflammation, placental insufficiency, and thrombosis. Anti-C5 antibody and C5aR antagonist peptides prevent aPL-mediated pregnancy loss and thrombosis in these experimental models. Clinical studies of anti-C5 monoclonal antibody in aPL-positive patients are limited to a small number of case reports. Ongoing and future clinical studies of complement inhibitors will help determine the role of complement inhibition in the management of aPL-positive patients.

## Abstract (tr)

Antifosfolipid sendromu (APS), ısrarcı antifosfolipid antikor (aPL) pozitifliği olan hastalarda görülen tromboz (arteriyel, venöz, küçük damar) ve/veya gebelik ile ilişkili morbidite ile karakterizedir. Hastalığın en şiddetli formu olan katastrofik APS, kısa süre içerisinde gelişen çoklu organ trombozları ile karakterizedir ve sıklıkla trombotik mikroanjiyopati (TMA) ile ilişkilidir. TMA geliştiren kompleman düzenleyici gen mutasyonları bulunan hastalarla benzer olarak, aPL-pozitif hastalardaki hiperkoagülopatide, endotel hücrelerinde artmış kompleman aktivasyonunun rolü vardır. APS’nin fare modellerinde, kompleman aktivasyonunun olması zorunludur ve kompleman (C) 5a ile reseptörü C5aR’nin etkileşmesi aPL-ile uyarılmış yangı, plasenta yetmezliği ve tromboza neden olur. Anti-C5 antikoru ve C5aR antagonisti olan peptidler bu deneysel modellerde, aPL-aracılı gebelik kaybı ve trombozu önler. aPL-pozitif hastalarda anti-C5 monoklonal antikorun klinik kullanımı az sayıdaki olgu sunumları ile sınırlıdır. Halen devam etmekte olan ve gelecekte yapılacak klinik çalışmalar, aPL-pozitif hastaların yönetiminde kompleman inhibitörlerinin rolünü belirlemede yardımcı olacaktır.

## INTRODUCTION

Antiphospholipid syndrome (APS) is characterized by thrombosis (arterial, venous, small vessel) and/or pregnancy morbidity occurring in patients with persistently positive antiphospholipid antibodies (aPL) [1]. The current treatment in APS focuses on final thrombosis rather than the initial aPL-induced prothrombotic and proinflammatory phenotypes. In parallel to our increased understanding of the mediators and mechanisms of the aPL-induced clinical events, the blockade of early pathogenic effects of aPL on target cells (monocytes, endothelial cells, or platelets) has been increasingly investigated.

The proposed mechanism of aPL-mediated thrombosis is the binding of aPL to endothelial cells [via β2-glycoprotein-I (β2GPI)] inducing a procoagulant state through different mechanisms including the expression of adhesion molecules and tissue factor (a physiologic initiator of coagulation and thrombin formation), and complement activation. In addition, products of complement activation, complement 3 (C3), C5a, and membrane attack complex (MAC), are potent mediators of platelet and endothelial cell activation; thus, the complement system is likely a critical step in the pathogenesis of APS [2].

Eculizumab, a humanized monoclonal antibody directed against C5, is approved for paroxysmal nocturnal hemoglobinuria (PNH) and atypical hemolytic uremic syndrome (aHUS) [3,4]. Given several recent case reports describing positive outcomes of severely ill aPL-positive patients treated with eculizumab, the purpose of this review is to discuss the importance of the complement system in the pathogenesis of APS, and the potential role of complement inhibition to prevent organ damage in aPL-positive patients.

## COMPLEMENT SYSTEM

**Figure 1:**
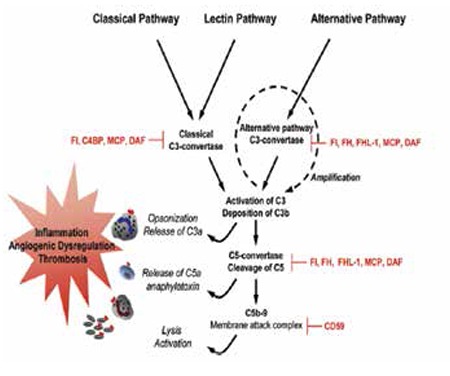
Human complement system. Three pathways are activated by immune complexes and apoptotic cells (classical); by microbes and stressors (lectin); and spontaneously (alternative). The effect of complement: clearance of apoptotic cells, opsonization of pathogens and immune complexes for phagocytosis, release of anaphylatoxins and lysis (shown in italics), and activation of effector cells that express receptors for C5a and/or C3a (neutrophils, monocytes, and platelets) are shown on the left. Complement inhibitors are indicated in red. Soluble inhibitors are factor I (FI), C4b-binding protein (C4BP), factor H (FH), and FH-like protein (FHL-1). Membrane-bound inhibitors include MCP (CD46), DAF (CD55), and CD59. Reprinted from Placenta 2010;31:561-567, Lynch AM, Salmon JE, Dysregulated complement activation as a common pathway of injury in preeclampsia and other pregnancy complications. Copyright (2015) with permission from Elsevier.

The complement system, composed of 30 proteins, protects the host against infections and initiates inflammation to kill microbes, remove dying cells, and dispose of immune complexes. The system is activated in a rapid fashion to opsonize or lyse a bacterium, while simultaneously triggering the release of proinflammatory and chemotactic peptides. The complement cascade can be triggered through 3 pathways: 1) the classical pathway, initiated by multivalent binding of the Fc fragments of antibody binding to the C1 complex; 2) the lectin pathway, binding specific sugars on a microbe to mannose binding lectin-associated proteases; and 3) the alternative pathway, spontaneous low-grade cleavage of C3 in plasma (Figure 1) [5,6].

These 3 pathways converge to generate C3 convertases, which cleave C3 into C3a and C3b. C3a is an anaphylatoxin that recruits and activates leukocyte effectors; C3b tags pathogens and immune complexes for opsonization. C3b leads to the assembly of C5 convertase and subsequent cleavage of C5 into C5a and C5b. C5a is a potent chemotactic molecule that recruits and stimulates leukocytes and endothelial cells, triggering release of cytokines/chemokines and the expression of adhesion molecules. Binding of C5b to cell surface assembles C5b-9 MAC, which inserts itself into membranes, damages cells, and activates proinflammatory pathways [5,6]. Furthermore, complement activation products contribute to thrombosis by augmenting the inflammatory responses of leukocytes and the endothelium, which in turn potentiate coagulation [6].

Factor B, factor D, and properdin contribute to the generation of C3b directly through the alternative pathway or through the amplification loop where C3b is formed. The production of C3b, triggered from engagement of the classical or lectin pathways, is augmented through the alternative pathway amplification [7].

Because of its potency, complement activation is regulated at each step. The major regulators of the alternate pathway amplification loop are plasma proteins complement factor H (CFH) and complement factor I (CFI), and a membrane cofactor protein (MCP) (CD46) [6].

## THROMBOTIC MICROANGIOPATHIES AND PAROXYSMAL NOCTURNAL HEMOGLOBINURIA

### Definitions

Thrombotic microangiopathy (TMA) is defined as thrombosis in arterioles and capillaries, which is commonly associated with thrombocytopenia, microangiopathic hemolytic anemia, and/or kidney failure [8]. Diseases associated with TMA are either hereditary or acquired; selected TMA syndromes are described in Table 1 [9]. Antiphospholipid antibody-positive patients, especially those with catastrophic APS [10], can develop TMA with or without medium-to-large vessel thrombosis (further discussed below).

Thrombotic thrombocytopenic purpura (TTP), which is hereditary (ADAMTS13 mutations) or acquired (antibodies against ADAMTS13), can present with a wide spectrum of manifestations including microangiopathic hemolytic anemia, thrombocytopenia, neurologic manifestations, gastrointestinal symptoms, purpura, and/or renal disease [9].

Hereditary [regulatory (CFH, CFI, or CD46) or effector (complement factor B or C3) gene mutations] or primary acquired (antibodies against factor H) complement-mediated TMA is due to the uncontrolled activation of the alternative pathway of the complement resulting in acute kidney injury and hypertension. These patients were previously classified as having “aHUS”, clinically defined as thrombocytopenia and microangiopathic hemolysis (with ADAMTS13 activity of >5% and a negative stool test for Shiga-toxin-producing infection) and one of the following: neurological symptoms, renal impairment, or gastrointestinal symptoms [9].

Hemolytic uremic syndrome (HUS) is a TMA featuring the triad of hemolytic anemia, thrombocytopenia, and acute renal impairment, mainly caused by Shiga-toxin-producing Escherichia coli. It is often preceded by bloody diarrhea (although one-third of the patients do not have bloody diarrhea), accounts for 90% of HUS cases in childhood, and does not relapse, and renal function recovers completely in >90% of cases. Traditionally, aHUS has been distinguished from HUS by the absence of diarrhea secondary to an Escherichia coli infection and a more severe clinical course [6]; however, rarely aHUS patients can present with gastrointestinal symptoms.

Several other conditions can be associated with secondary TMA (Table 1). Disseminated intravascular coagulation is associated with intravascular activation and consumption of the different coagulation system components depending on the underlying cause. Preeclampsia is the onset of hypertension and proteinuria after 20 weeks of gestation; disease manifestations range from mild blood pressure elevations to severe hypertension, the HELLP syndrome (hemolysis, elevated liver enzymes, and low platelets), and eclampsia (seizures).

PNH is a disease of hematopoietic stem cells resulting in intravascular hemolysis, hemoglobinuria, and thromboembolism due to the deficiency of two proteins (CD55-decay accelerating factor; CD59-membrane inhibitor of reactive lysis) that inhibit the activation and cytolytic functions of the complement system [11].

### Complement System in Thrombotic Microangiopathies and Paroxysmal Nocturnal Hemoglobinuria

Mouse studies demonstrate that the absence of complement regulatory proteins is associated with TMA and pregnancy loss [12]. As discussed above, mutations in complement regulatory proteins result in aHUS and PNH, both associated with microvascular endothelial cell activation, cell injury, and thrombosis [13].

Furthermore, a relationship exists between activation of the complement system and development of an imbalance in angiogenic factors. Mouse models show that C5a induces release of antiangiogenic factors [14] and complement deletion prevents placental insufficiency in mouse models of preeclampsia [15]. Excess production of an antiangiogenic factor by the placenta and inflammatory cells leads to impaired placental development and placental dysfunction [5,14,16]. In normal pregnancies, excessive complement activation is prevented by complement regulatory proteins that are highly expressed on trophoblast membranes (CD55 and CD59) and circulating complement regulatory proteins (CFH, CFI, and C4 binding protein). In preeclampsia, complement activation products (C4d and C5b-9) are detected on trophoblasts, and in vivo hypoxia enhances MAC deposition on villous trophoblasts [17].

### Complement Inhibition in Thrombotic Microangiopathies and Paroxysmal Nocturnal Hemoglobinuria

Eculizumab is a recombinant humanized monoclonal antibody that binds to the terminal complement protein C5, inhibits its cleavage into C5a and C5b, and prevents the generation of MAC. In PNH patients, eculizumab reduces the frequency of hemolysis, hemoglobinuria, transfusion, and thrombosis [18]. In aHUS patients, eculizumab inhibits complement-mediated TMA (resolving thrombocytopenia and TMA) and improves renal transplantation outcomes by allowing plasma exchange-dependent patients to stop this treatment [4]. Eculizumab has been also used off-label in TTP patients refractory to plasmapheresis [19].

## ANTIPHOSPHOLIPID SYNDROME

### Definitions

Antiphospholipid antibodie are a heterogeneous family of autoantibodies directed against phospholipid-binding plasma proteins, most commonly β2GPI. The predominant hypothesis regarding the origin of aPL states that an incidental exposure to environmental agents with β2GPI-like peptides induces aPL in susceptible individuals (molecular mimicry) [20].

A positive aPL test is not always “clinically significant”; transient aPL positivity is not uncommon, especially during infections [21]. Documentation of aPL persistence is therefore important. High titers of anticardiolipin antibodies (aCL) and aβ2GPI as well as IgG/M isotypes are more concerning than lower titers and IgA isotypes. Whereas a positive lupus anticoagulant (LA) test is a better predictor of thrombosis than aCL and aβ2GPI, false-positive and false-negative LA tests can occur for patients on anticoagulation [22]. Documentation of a positive LA test requires 4 criteria according to International Society of Thrombosis and Haemostasis guidelines [23]: 1) demonstration of a prolonged phospholipid-dependent coagulation screening test, such as activated partial thromboplastin time or dilute Russell viper venom time; 2) failure to correct the prolonged screening test by mixing the patient’s plasma with normal platelet-poor plasma, demonstrating the presence of an inhibitor; 3) shortening or correction of the prolonged screening test by the addition of excess phospholipid, demonstrating phospholipid dependency; and 4) exclusion of other inhibitors.

Our definition of a “clinically significant” aPL profile is: 1) LA test positivity in accordance with the above guidelines [23]; 2) aCL IgG/M of ≥40 U [1]; and/or 3) aβ2GPI IgG/M of ≥40 U tested twice at least 12 weeks apart. Clinical judgment is required to determine thrombosis risk when aPL results are equivocal, e.g., LA test not measurable because the patient is anticoagulated, aCL or aβ2GPI IgG/M titers are between 20 and 39 U, and/or aCL or aβ2GPI IgA is the only positive aPL test.

In patients with clinically significant aPL profiles, clinical manifestations may be varied: 1) asymptomatic aPL positivity (no history of thrombosis or pregnancy morbidity); 2) non-criteria manifestations of aPL, e.g., livedo reticularis, thrombocytopenia, hemolytic anemia, cardiac valve disease, aPL-associated nephropathy, or cognitive dysfunction; 3) pregnancy morbidity (recurrent embryonic or fetal loss, preeclampsia, placental insufficiency, and fetal growth restriction); 4) venous, arterial, or small vessel thrombosis (stroke is the most common presentation of arterial thrombosis; deep vein thrombosis is the most common venous manifestation); and 5) catastrophic APS (multiple organ thrombosis developing in a short period), which is usually associated with TMA [1,10].

### Mechanisms of Antiphospholipid Antibody-Mediated Injury

Antiphospholipid antibodies induce thrombosis and placental injury through multiple mechanisms. The process begins with activation or apoptosis of platelets, endothelial cells, or trophoblasts, during which phosphatidylserine (a negatively charged phospholipid) migrates from the inner to the normally electrically neutral outer cell membrane. β2GPI, which potentially exists in the circulation in a circular form [24], then binds to PS independently of aβ2GPI (via β2GPI surface receptors such as apoER2′, annexin A2, or a toll-like receptor). After this binding, the circular β2GPI opens up to expose domain I and aPL binds to β2GPI, directly stimulating cells through surface receptors. Antiphospholipid antibodies can also stimulate cells indirectly by activating the classic complement pathway. The generation of C5a induces expression of adhesion molecules and tissue factor and activation of monocytes, polymorphonuclear cells, and platelets, and it triggers the release of proinflammatory mediators (e.g., tumor necrosis factor, vascular endothelial growth factor receptor-1) and initiation of the proadhesive and prothrombotic state. Thus, multiple pathways are engaged by aPL binding to the surface. Crosslinking apoER2′ receptors antagonize endothelial nitric oxide synthase, blocking its phosphorylation and leading to increased leukocyte adhesion and thrombosis. Both nuclear factor κB and p38 mitogen-activated protein kinase play a role in the intracellular signaling cascade. Antiphospholipid antibodies also downregulate the expression of trophoblast signal transducer and activator of transcription 5 (STAT5), reducing the endometrial stromal cell production of prolactin (PRL) and insulin growth factor binding protein-1 (IGFBP-1) [25].

Patients with aPL may have thrombocytopenia, and its mechanisms include: 1) promoting platelet activation and aggregation; 2) antiplatelet antibodies directed against platelet membrane glycoproteins such as aGPIIb/IIIa [26]; and 3) platelet destruction as seen in TMA, including catastrophic APS.

Thrombotic microangiopathy, which is common in catastrophic APS and/or transplant rejection, is the most characteristic lesion of APS nephropathy; the pathologic changes may be similar to other TMAs, e.g., HUS, TTP, and preeclampsia. In addition, aPL-nephropathy patients can develop chronic cortical ischemia/infarction (arteriosclerosis, arteriolosclerosis, arterial fibrous intimal hyperplasia, glomerular ischemia, interstitial fibrosis, tubular thyroidization, tubular atrophy, and/or organized thrombi with/without recanalization) [1,27,28]. Recently, it has been shown that in APS patients these vascular renal lesions are associated with the activation of the mammalian target of rapamycin pathway [29]. Given the tissue damage prominent in renal TMA, it is likely that inflammatory damage by recruited leukocytes and vascular cell activation are amplified by complement activation products generated as a consequence of the alternative pathway.

### Complement System in Antiphospholipid Antibody-Mediated Injury

Passive transfer of human aPL results in endothelial cell activation and pregnancy loss in animal models [30,31]. Endothelial cell activation correlates with a prothrombotic phenotype in vitro and enhances thrombus formation in vivo [30,32]. Complement activation, specifically C5, is a necessary intermediary event in both thrombosis and pregnancy complications associated with aPL in rodent models [33].

Complement activation initiates and amplifies the cellular features characteristic of APS: endothelial cell activation, monocyte tissue factor expression, and platelet aggregation. Generation of C5a contributes to vascular inflammation [34,35]. Complement 5a interacts with its receptor, C5aR, to promote recruitment and activation of neutrophils (PMNs) and monocytes, and activation of EC [29]. C5a-C5aR ligation also: 1) upregulates neutrophil-derived TF expression, thought to be one mechanism of aPL-mediated coagulation and disseminated thrombosis [36]; 2) leads to trophoblast injury and angiogenic factor imbalance in aPL-induced fetal injury [37]; and 3) produces lesions such as those seen in TMA in mouse models.

Mice deficient in complement components C3, C5, C6, or C5a receptors are resistant to aPL-induced enhanced thrombophilia and endothelial cell activation [38]. Treatment with anti-C5 monoclonal antibody or C5aR antagonist peptides attenuates thrombosis in mouse models of APS [31]. In mouse models of surgically induced thrombus formation, complement activation plays an important role in the increased thrombosis and adhesion of leukocytes to endothelial cells caused by treatment with aPL. Heparin has anticomplement effects, as well as acting as an anticoagulant, which may explain some of its efficacy in APS [39].

In addition, mouse models of aPL-induced pregnancy loss and growth restriction show that C4, factor B, C3, C5, and C5aR are required for placental injury [30]. Complement deposition is present in human placenta from patients with APS [40]. Patients with aPL, with or without clinical manifestation of APS, show elevated circulating levels of Bb and C3a fragments [41]; the fact that the complement contributes to placental injury is suggested by the evidence for C4d on trophoblasts in patients with APS [42]. In a recent study, APS patients were found to have elevated C3a levels in plasma, but there was no correlation with the development of thrombosis [43].

### Complement Inhibition in Antiphospholipid Antibody-Positive Patients

Clinical studies of anti-C5 monoclonal antibody (eculizumab) in aPL-positive patients are limited to a small number of case reports.

The first report was published in 2010 by Lonze et al. describing improvement of TMA after kidney transplant in an eculizumab-treated patient with a history of catastrophic aPL syndrome [44]. Of note, the patient also received systemic anticoagulation and standard immunosuppression. Velik-Salchner et al. questioned the effectiveness of eculizumab in this patient by drawing attention to the ability of heparin to inhibit complement in APS mouse models and the possibility of TMA in aPL-positive patients that does not involve complement activation [45].

In 2011, Hadaya et al. reported an aPL-positive systemic lupus erythematosus patient who underwent a living-related kidney transplantation, which was complicated by recurrent thrombotic microangiopathy [46]. Despite the standard posttransplantation regimen and daily plasma exchange, renal function did not improve. The patient received 5 weekly infusions of eculizumab, and the renal function normalized after 6 months. Darnige et al. studied aPL titers in 20 PNH patients receiving eculizumab [47]. Only 3 patients had preinfusion low-titer aCL or aβ2GPI (negative lupus anticoagulant); there was no significant change in the titers after 11 weeks of treatment.

In 2012, Shapira et al. reported a catastrophic APS patient resistant to anticoagulation, immunosuppression, plasmapheresis, and rituximab; eculizumab successfully blocked complement activity, aborted progressive thrombosis, and reversed thrombocytopenia [48].

In 2013, Canaud et al. reported 3 aPL-nephropathy patients treated with eculizumab following TMA after kidney transplantation due to aPL-nephropathy recurrence [49]. Based on pre- and posttransplantation biopsies, the investigators showed that: 1) eculizumab results in remission in plasmapheresis-resistant thrombotic angiopathy related to aPL-nephropathy recurrence; 2) persistent C5b-9 deposition is commonly found in allografts developing posttransplant thrombotic microangiopathy; and 3) chronic vascular changes related to aPL may not be related to complement activation. In the same year, another case report [50] and personal communications during the 14th International Congress on aPL described catastrophic APS patients who failed to respond to eculizumab [51].

In 2014, several case reports or series described the outcomes of eculizumab-treated aPL-positive patients: 1) Bakhtar et al. described a lupus and APS patient who developed biopsy-proven TMA, thrombocytopenia, and hemolysis 3 years after living-related kidney transplantation; after 7 months of eculizumab, there was no evidence of TMA on biopsy and both hemoglobin and platelets were normal [52]; 2) Lonze et al. reported 3 APS patients (2 with catastrophic APS, and including the follow-up information of the first eculizumab-receiving patient reported in 2010) who were treated with anticoagulation and eculizumab prior to and following live donor renal transplantation (2 also received plasmapheresis); after a follow-up ranging from 4 months to 4 years, all patients had functioning renal allografts [53]; 3) Strakhan et al. reported another catastrophic APS patient (multiple strokes, non-ST elevation myocardial infarction, end-stage renal disease due to TMA, intraretinal hemorrhage, and thrombocytopenia) who had no response to corticosteroids and plasma exchange (no heparin during the acute period); the patient’s condition stabilized after eculizumab [54]; 4) Zapantis et al. reported 3 APS patients with recurrent thrombosis and thrombocytopenia unresponsive to conventional therapy with significant improvement of thrombocytopenia after eculizumab administration (personal communication) [55].

Given the above reports, complement inhibition may have a role as an adjuvant or main therapy for APS patients refractory to anticoagulation; however, publication bias is a concern as well as the lack of systematic clinical studies. Thus, more mechanistic and clinical studies are needed before eculizumab can be recommended [50]. Clinicians should keep in mind that the infection risk of eculizumab is mainly with encapsulated organisms, specifically meningococcal. Patients must be immunized against Neisseria meningitidis before treatment with eculizumab.

### Ongoing Observational and Interventional Complement-Related Clinical Studies in Antiphospholipid Antibody-Positive Patients

Potential novel approaches to target terminal complement activation include C5aR antagonists (antibodies or peptides) and soluble and targeted complement regulatory proteins.

The PROMISE Study (Predictors of pRegnancy Outcome: bioMarkers In APS and Systemic lupus Erythematosus) (clinicaltrials.gov#: NCT00198068), a prospective, multicenter observational study, aims to translate findings in mice to humans and evaluate the role of complement in lupus and aPL-associated pregnancy complications. The study, which has recruited over 700 patients as of December 2014, is ongoing and will test the hypotheses that classical, alternative, and terminal complement pathway activation and dysregulation of angiogenic factors will be detected in the circulation of patients destined for pregnancy complications. The PROMISE Study has the potential to identify new biomarkers for adverse pregnancy outcomes that in addition to being good predictors of these outcomes are also part of the mechanistic process of these pregnancy complications. In the future, it may be possible to identify those patients in whom complement inhibitors are likely to prevent or modify the inflammatory-related sequelae associated with adverse pregnancy outcomes.

One open-label interventional phase II prevention trial (clinicaltrials.gov#: NCT01029587) is investigating whether blocking the complement cascade with eculizumab in 10 patients with a prior history of catastrophic APS who are undergoing kidney transplant will lead to increased transplant success. Three patients included in the protocol have already been reported [44,51] and the estimated completion date was August 2015.

Another open-label multicenter international phase IIa treatment trial (clinicaltrials.gov#: NCT02128269) is evaluating the safety and tolerability of an intravenous C5a inhibitor in persistently aPL-positive patients with at least one of the following non-criteria manifestations of APS: aPL-nephropathy, skin ulcers, and/or thrombocytopenia.

## CONCLUSION

Animal and human studies have confirmed the relevance of complement inhibition in many inflammatory and microthrombotic diseases including APS. Thus, complement inhibition may have a role for APS patients refractory to anticoagulation; however, more clinical data are needed. Future mechanistic and clinical studies of eculizumab and other complement inhibitors will be necessary to individualize treatment. We hope that the results from the ongoing studies will be available for further discussion at the 15th International Congress on aPL (İstanbul, Turkey) (www.apsistanbul2016.org).

## Figures and Tables

**Table 1:**
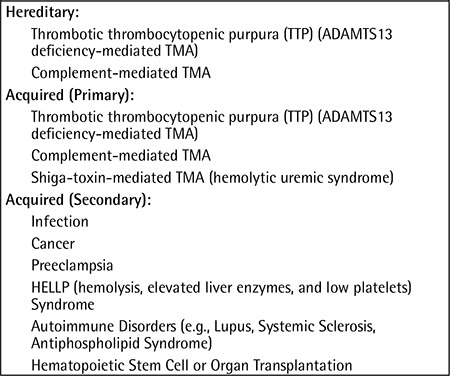
Hereditary and acquired thrombotic microangiopathies (adapted from George and Nester [9]).

## References

[1] Miyakis S, Lockshin MD, Atsumi T, Branch DW, Brey RL, Cervera R, Derksen RH, DE Groot PG, Koike T, Meroni PL, Reber G, Shoenfeld Y, Tincani A, Vlachoyiannopoulos PG, Krilis SA (2006). International consensus statement on an update of the classification criteria for definite antiphospholipid syndrome (APS). J Thromb Haemost.

[2] Girardi G, Redecha P, Salmon JE (2004). Heparin prevents antiphospholipid antibody-induced fetal loss by inhibiting complement activation. Nat Med.

[3] Hillmen P, Young NS, Schubert J, Brodsky RA, Socié G, Muus P, Röth A, Szer J, Elebute MO, Nakamura R, Browne P, Risitano AM, Hill A, Schrezenmeier H, Fu CL, Maciejewski J, Rollins SA, Mojcik CF, Rother RP, Luzzatto L (2006). The complement inhibitor eculizumab in paroxysmal nocturnal hemoglobinuria. N Engl J Med.

[4] Legendre CM, Licht C, Muus P, Greenbaum LA, Babu S, Bedrosian C, Bingham C, Cohen DJ, Delmas Y, Douglas K, Eitner F, Feldkamp T, Fouque D, Furman RR, Gaber O, Herthelius M, Hourmant M, Karpman D, Lebranchu Y, Mariat C, Menne J, Moulin B, Nürnberger J, Ogawa M, Remuzzi G, Richard T, Sberro-Soussan R, Severino B, Sheerin NS, Trivelli A, Zimmerhackl LB, Goodship T, Loirat C (2013). Terminal complement inhibitor eculizumab in atypical hemolytic-uremic syndrome. N Engl J Med.

[5] Lynch AM, Salmon JE (2010). Dysregulated complement activation as a common pathway of injury in preeclampsia and other pregnancy complications. Placenta.

[6] Java A, Atkinson J, Salmon J (2013). Defective complement inhibitory function predisposes to renal disease. Annu Rev Med.

[7] Holers VM (2008). The spectrum of complement alternative pathway-mediated diseases. Immunol Rev.

[8] Benz K, Amann K (2010). Thrombotic microangiopathy: new insights. Curr Opin Nephrol Hypertens.

[9] George JN, Nester CM (2014). Syndromes of thrombotic microangiopathy. N Engl J Med.

[10] Aguiar CL, Erkan D (2013). Catastrophic antiphospholipid syndrome: how to diagnose a rare but highly fatal disease. Ther Adv Musculoskeletal Dis.

[11] Parker JC (2012). Paroxysmal nocturnal hemoglobinuria. Curr Opin Hematol.

[12] Xu C, Mao D, Holers VM, Palanca B, Cheng AM, Molina H (2000). A critical role for murine complement regulator crry in fetomaternal tolerance. Science.

[13] Hillmen P, Young NS, Schubert J, Brodsky RA, Socié G, Muus P, Röth A, Szer J, Elebute MO, Nakamura R, Browne P, Risitano AM, Hill A, Schrezenmeier H, Fu CL, Maciejewski J, Rollins SA, Mojcik CF, Rother RP, Luzzatto L (2006). The complement inhibitor eculizumab in paroxysmal nocturnal hemoglobinuria. N Engl J Med.

[14] Girardi G, Yarilin D, Thurman JM, Holers VM, Salmon JE (2006). Complement activation induces dysregulation of angiogenic factors and causes fetal rejection and growth restriction. J Exp Med.

[15] Gelber SE, Brent E, Redecha P, Perino G, Tomlinson S, Davisson RL, Salmon JE (2015). Prevention of defective placentation and pregnancy loss by blocking innate immune pathways in a syngeneic model of placental insufficiency. J Immunol.

[16] Lynch AM, Murphy JR, Byers T, Gibbs RS, Neville MC, Giclas PC, Salmon JE, Holers VM (2008). Alternative complement pathway activation fragment Bb in early pregnancy as a predictor of preeclampsia. Am J Obstet Gynecol.

[17] Rampersad R, Barton A, Sadovsky Y, Nelson DM (2008). The C5b-9 membrane attack complex of complement activation localizes to villous trophoblast injury in vivo and modulates human trophoblast function in vitro. Placenta.

[18] Hillmen P, Hall C, Marsh JC, Elebute M, Bombara MP, Petro BE, Cullen MJ, Richards SJ, Rollins SA, Mojcik CF, Rother RP (2004). Effect of eculizumab on hemolysis and transfusion requirements in patients with paroxysmal nocturnal hemoglobinuria. N Engl J Med.

[19] Tsai E, Chapin J, Laurence JC, Tsai HM (2013). Use of eculizumab in the treatment of a case of refractory, ADAMTS13-deficient thrombotic thrombocytopenic purpura: additional data and clinical follow-up. Br J Haematol.

[20] Shoenfeld Y (2003). Etiology and pathogenetic mechanisms of the antiphospholipid syndrome unraveled. Trends Immunol.

[21] Avcin T, Toplak N (2007). Antiphospholipid antibodies in response to infection. Curr Rheumatol Rep.

[22] Galli M, Luciani D, Bertolini G, Barbui T (2003). Lupus anticoagulants are stronger risk factors for thrombosis than anticardiolipin antibodies in the antiphospholipid syndrome: a systematic review of the literature. Blood.

[23] Pengo V, Tripodi A, Reber G, Rand JH, Ortel TL, Galli M (2009). Update of the guidelines for lupus anticoagulant detection. Subcommittee on Lupus Anticoagulant/Antiphospholipid Antibody of the Scientific and Standardisation Committee of the International Society on Thrombosis and Haemostasis. J Thromb Haemost.

[24] Giannakopoulos B, Krilis SA (2013). The pathogenesis of the antiphospholipid syndrome. N Engl J Med.

[25] Erkan D, Salmon J, Lockshin MD, Firestein GS, Budd RC, Gabriel SE, McInnes IB, Odell JR (2013). Antiphospholipid syndrome. Kelley’s Textbook of Rheumatology, 9th ed.

[26] Alpert D, Mandl LA, Erkan D, Yin W, Peersche EI, Salmon JE (2008). Anti-heparin platelet factor 4 antibodies in systemic lupus erythematosus are associated with IgM antiphospholipid antibodies and the antiphospholipid syndrome. Ann Rheum Dis.

[27] D’Cruz D (2009). Renal manifestations of the antiphospholipid syndrome. Curr Rheumatol Rep.

[28] Gigante A, Gasperini ML, Cianci R, Barbano B, Giannakakis K, Donato D, Fuiano G, Amoroso A (2009). Antiphospholipid antibodies and renal involvement. Am J Nephrol.

[29] Canaud G, Bienaimé F, Tabarin F, Bataillon G, Seilhean D, Noël LH, Dragon-Durey MA, Snanoudj R, Friedlander G, Halbwachs-Mecarelli L, Legendre C, Terzi F (2014). Inhibition of the mTORC pathway in the antiphospholipid syndrome. N Engl J Med.

[30] Girardi G, Berman J, Redecha P, Spruce L, Thurman JM, Kraus D, Hollmann TJ, Casali P, Caroll MC, Wetsel RA, Lambris JD, Holers VM, Salmon JE (2003). Complement C5a receptors and neutrophils mediate fetal injury in the antiphospholipid syndrome. J Clin Invest.

[31] Pierangeli SS, Colden-Stanfield M, Liu X, Barker JH, Anderson GL, Harris EN (1999). Antiphospholipid antibodies from antiphospholipid syndrome patients activate endothelial cells in vitro and in vivo. Circulation.

[32] Simantov R, LaSala JM, Lo SK, Gharavi AE, Sammaritano LR, Salmon JE, Silverstein RL (1995). Activation of cultured vascular endothelial cells by antiphospholipid antibodies. J Clin Invest.

[33] Bulla R, Bossi F, Fischetti F, De Seta F, Tedesco F (2005). The complement system at the fetomaternal interface. Chem Immunol Allergy.

[34] Giannakopoulos B, Passam F, Rahgozar S, Krilis SA (2007). Current concepts on the pathogenesis of the antiphospholipid syndrome. Blood.

[35] Peerschke EI, Yin W, Ghebrehiwet B (2010). Complement activation on platelets: implications for vascular inflammation and thrombosis. Mol Immunol.

[36] Ritis K, Doumas M, Mastellos D, Micheli A, Giaglis S, Magotti P, Rafail S, Kartalis G, Sideras P, Lambris JD (2006). A novel C5a receptor-tissue factor crosstalk in neutrophils links innate immunity to coagulation pathways. J Immunol.

[37] Redecha P, Tilley R, Tencati M, Salmon JE, Kirchhofer D, Mackman N, Girardi G (2007). Tissue factor: a link between C5a and neutrophil activation in antiphospholipid antibody induced fetal injury. Blood.

[38] Pierangeli SS, Girardi G, Vega-Ostertag M, Liu X, Espinola RG, Salmon J (2005). Requirement of activation of complement C3 and C5 for antiphospholipid antibody-mediated thrombophilia. Arthritis Rheum.

[39] Licht C, Fremeaux-Bacchi V (2009). Hereditary and acquired complement dysregulation in membranoproliferative glomerulonephritis. Thromb Haemost.

[40] Shamonki JM, Salmon JE, Hyjek E, Baergen RN (2007). Excessive complement activation is associated with placental injury in patients with antiphospholipid antibodies. Am J Obstet Gynecol.

[41] Oku K, Amengual O, Atsumi T (2012). Pathophysiology of thrombosis and pregnancy morbidity in the antiphospholipid syndrome. Eur J Clin Invest.

[42] Viall CA, Chamley LW (2014). Histopathology in the placentae of women with antiphospholipid antibodies: a systematic review of the literature. Autoimmun Rev.

[43] Devreese K, Hoylaerts MF (2010). Is there an association between complement activation and antiphospholipid antibody related thrombosis?. Thromb Haemost.

[44] Lonze BE, Singer AL, Montgomery RA (2010). Eculizumab and renal transplantation in a patient with CAPS. N Engl J Med.

[45] Velik-Salchner C, Lederer W, Wiedermann F (2011). Eculizumab and renal transplantation in a patient with catastrophic antiphospholipid syndrome: effect of heparin on complement activation. Lupus.

[46] Hadaya K, Ferrari-Lacraz S, Fumeaux D, Boehlen F, Toso C, Moll S, Martin PY, Villard J (2011). Eculizumab in acute recurrence of thrombotic microangiopathy after renal transplantation. Am J Transplant.

[47] Darnige L, Zemori L, Socié G, Fischer AM (2011). Antiphospholipid antibodies in patients with paroxysmal nocturnal haemoglobinuria receiving eculizumab. Br J Haematol.

[48] Shapira I, Andrade D, Allen SL, Salmon JE (2012). Brief report: induction of sustained remission in recurrent catastrophic antiphospholipid syndrome via inhibition of terminal complement with eculizumab. Arthritis Rheum.

[49] Canaud G, Kamar N, Anglicheau D, Esposito L, Rabant M, Noël LH, Guilbeau-Frugier C, Sberro-Soussan R, Del Bello A, Martinez F, Zuber J, Rostaing L, Legendre C (2013). Eculizumab improves posttransplant thrombotic microangiopathy due to antiphospholipid syndrome recurrence but fails to prevent chronic vascular changes. Am J Transplant.

[50] Mushin SA, Khianey R, Erkan D (2013). Discordant aPTT and anti-FXa values in a catastrophic antiphospholipid syndrome patient receiving intravenous unfractionated heparin. In: 14th International Congress on Antiphospholipid Antibodies Abstract Book.

[51] Erkan D, Aguiar CL, Andrade D, Cohen H, Cuadrado MJ, Danowski A, Levy RA, Ortel TL, Rahman A, Salmon JE, Tektonidou MG, Willis R1, Lockshin MD (2014). 14th International Congress on Antiphospholipid Antibodies: Task force report on antiphospholipid syndrome treatment trends. Autoimmun Rev.

[52] Bakhtar O, Thajudeen B, Braunhut BL, Yost SE, Bracamonte ER, Sussman AN, Kaplan B (2014). A case of thrombotic microangiopathy associated with antiphospholipid antibody syndrome successfully treated with eculizumab. Transplantation.

[53] Lonze BE, Zachary AA, Magro CM, Desai NM, Orandi BJ, Dagher NN, Singer AL, Carter-Monroe N, Nazarian SM, Segev DL, Streiff  MB, Montgomery RA (2014). Eculizumab prevents recurrent antiphospholipid antibody syndrome and enables successful renal transplantation. Am J Transplant.

[54] Strakhan M, Hurtado-Sbordoni M, Galeas N, Bakirhan K, Alexis K, Elrafei T (2014). 36-year-old female with catastrophic antiphospholipid syndrome treated with eculizumab: a case report and review of literature. Case Rep Hematol.

[55] Zapantis E, Furie R, Horowitz D (2014). Eculizumab in antiphospholipid antibody syndrome. Arthritis Rheum.

